# Dry Cold for Piles

**Published:** 1875-10

**Authors:** 


### DRY COLD FOR PILES.

Dr. Mattson's syphon-cone, by the aid of which intense cold is readily applied for the speedy and permanent relief of this distressing disease, can at last be found in the vicinity of our office. We have spoken of its great merits heretofore, and our allusions to it have brought us letters and calls from a number of great sufferers. We did not know where to send them for the instrument. Now the Messrs. Hegeman—one of whose stores is on the corner of Broadway and Eighth Street—have laid in a supply of these valuable devices, and there our friends can apply. The cost is $3.50 we believe, and to any one who suffers from the burning agony attending this complaint, it will pay for itself in the first ten minutes. It is as simple as may be. A single squeeze of the bulb establishes the syphon-current, and once begun it keeps up till the water supply is exhausted. The moment the dry chill strikes the inflamed, engorged, irritated, or fissured parts, all pain and annoyance cease, and the brain is soothed into a delicious calm. In ordinary cases a full cure comes from the use of the cone a few days. Why every druggist does not keep this valuable instrument we are at a loss to determine. The trade is supplied by any of the special houses of the Mattson Manufacturing Co., in New York, Philadelphia, Boston, Cincinnati, Chicago, St. Louis, and San Francisco, and also in London and Paris, as well as by all wholesale dealers in druggist's sundries and fancy rubber goods. We hope to hear no more complaints that the article can not be found.

